# Comparative Transcriptomic Analysis Reveals New Insights into Spawn Aging in *Agaricus bisporus*: Mitochondrial Dysfunction

**DOI:** 10.3390/ijms26020849

**Published:** 2025-01-20

**Authors:** Lili Shu, Zhiheng Zeng, Meiyuan Chen, Jiazhi Zhao, Xiaoyan Zhang, Jianqing Dai, Zhixin Cai, Yuanping Lu, Zhiheng Qiu, Hui Zeng

**Affiliations:** 1 Institute of Edible Mushroom, Fujian Academy of Agricultural Sciences, National-Local Joint Engineering Research Center for Breeding and Cultivation of Featured Edible Mushroom, Fuzhou 350011, China; 2017500023@syau.edu.cn (L.S.); zengzhiheng@faas.cn (Z.Z.); chenmeiyuan@faas.cn (M.C.); daijianqing@faas.cn (J.D.); caizhixin76@163.com (Z.C.); yuanplu1106@163.com (Y.L.); 2 Modern Protected Horticulture Engineering & Technology Center, College of Horticulture, Shenyang Agricultural University, Shenyang 110866, China; jiazhi@stu.syau.edu.cn (J.Z.); zhangxiaoyan199809@163.com (X.Z.)

**Keywords:** *Agaricus bisporus*, spawn aging, mitochondrial dysfunction, comparative transcriptome, molecular mechanisms

## Abstract

Spawn aging poses a substantial challenge to the *Agaricus bisporus* industry. This study focuses on the role of mitochondrial dysfunction in the aging process of *A. bisporus* spawn. We conducted a comprehensive comparative transcriptome analysis to elucidate the molecular mechanisms underlying *A. bisporus* spawn aging. A total of 1620 genes with significant expression changes between the normal and aged spawn were identified, including 917 up-regulated genes and 703 down-regulated genes. Our results revealed a notable down-regulation of genes involved in carbohydrate metabolism, mitochondrial energy metabolism, reactive oxygen species (ROS) scavenging, repair mechanisms for oxidative stress-induced damage, fatty acid β-oxidation, and amino acid degradation in aged *A. bisporus* spawn. Additionally, we observed a decreased expression of genes involved in critical signal transduction pathways associated with mitochondrial function in aged mycelium as well as genes responsible for maintaining mitochondrial stability. The up-regulated genes in aged spawn mainly affect mitochondrial fission and programmed cell death, impacting mitochondrial function. Overall, the present study first provides evidence for the pivotal role of mitochondrial dysfunction in the aging process of *A. bisporus* spawn and contributes to the development of targeted strategies to enhance mitochondrial function, mitigate spawn aging, and improve the yield and quality of *A. bisporus* cultivation.

## 1. Introduction

*Agaricus bisporus*, commonly known as the button mushroom, plays a significant role in the global economy and diet, offering not only nutritional value but also medicinal benefits [1]. As the most widely cultivated edible mushroom, its production and quality control are of paramount importance to agricultural and food industries worldwide. Among the various challenges faced in mushroom cultivation, spawn aging is a crucial but insufficiently investigated factor that significantly impacts both the yield and quality [2]. Spawn aging refers to the gradual decline in the vitality and growth potential of mushroom spawn over time, which can lead to significant economic losses. The underlying mechanisms of spawn aging in *A. bisporus* are complex and have been the subject of limited research. Therefore, understanding these bases is crucial for developing strategies to enhance spawn longevity, improve mushroom quality, and increase production efficiency.

The quality and vitality of spawn play a crucial role in mushroom production [3]. However, its aging process, characterized by a gradual decline in vigor and reproductive capacity over time, presents a significant challenge to growers. The spawn of *A. bisporus*, particularly wheat seed spawn, is prone to exude yellow water after aging. This secretion is accompanied by an unpleasant odor and reduced viability. Furthermore, the growth rate of mycelia experiences a significant deceleration, leading to a production decrease ranging from 20% to 50%, and, in severe cases, complete absence of mushroom formation [2]. Fungal aging is a relatively slow, time-dependent process [4]. The phenomenon of fungal aging is commonly observed as the cultivation period progresses [5]. Despite its significance, the biological mechanisms underlying fungal aging remain poorly comprehended, primarily due to the intricate nature of the fungal life cycle and the complex interplay between genetic and environmental factors that impact aging [6].

The current focus of aging research primarily lies in animal cells, while studies on fungal aging predominantly center around model fungi such as *Podospora anserina* and *Neurospora* [5,7]. Aging is a complex process governed by an intricate network of mechanisms that collectively contribute to the gradual decline in physiological function and increased susceptibility to diseases. At the molecular level, aging is characterized by the accumulation of DNA damage resulting from replication errors, environmental insults, and inefficient repair mechanisms over time [8]. This accumulation disrupts cellular homeostasis and can activate senescence pathways, effectively halting cell division and altering intercellular communication [9]. Additionally, the process of aging is also characterized by a progressive decline in proteostasis, which encompasses the cell’s diminishing capacity to accurately synthesize, fold, and degrade proteins. This loss results in misfolded protein accumulation, further impairing cellular function [10]. Mitochondrial dysfunction also plays a critical role in aging as mitochondria are key regulators of energy production and apoptosis. Several factors have been shown to trigger the decline in mitochondrial function during the aging process, including increased production of ROS, mutations in mitochondrial DNA (mtDNA), protein oxidation, abnormalities in energy metabolism, and a reduction in mitochondrial biogenesis [11,12]. Together, these mechanisms underscore the complexity of aging, highlighting the challenge of unraveling its underlying biological processes. A previous study found that the disruption of mitochondrial function in fungi can lead to increased aging of mycelium [5]. The vitality of mycelium will directly affect spawn quality. However, there are no reports on the relationship between mitochondrial dysfunction and aging of edible fungi spawn.

This study aims to fill this gap by employing high-throughput transcriptome analysis to explore the role of mitochondrial dysfunction in spawn aging of *A. bisporus*. Through comprehensive examination of gene expression profiles in normal and aged mushroom spawn, our objective is to identify key genes, pathways, and regulatory networks involved in the aging process. Specifically, we focus on unraveling the molecular drivers of reduced spawn vitality and exploring the biological pathways that are differentially affected during aging. This research is expected to provide novel insights into the molecular basis of spawn aging, offering potential targets for genetic and biotechnological interventions to extend spawn shelf life and improve mushroom production.

## 2. Results

### 2.1. Aging Altered the Morphology of A. bisporus Mycelia

**Figure 1:**
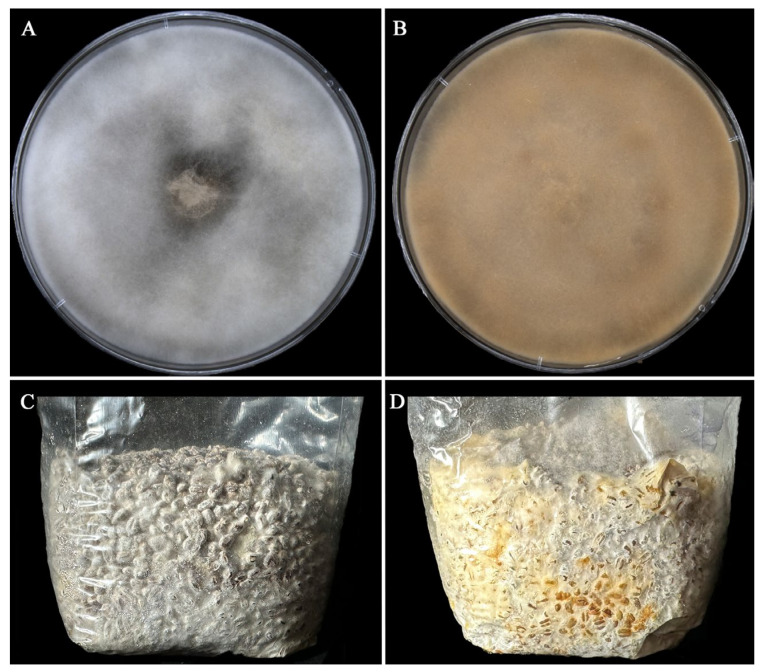
Morphological characteristics of normal As2796 and aged As2796Y strains in PDA and WGM medium. (**A**) Normal As2797 mycelia grew on PDA medium. (**B**) The aged As2796Y mycelia grew on PDA medium. (**C**) Normal As2797 mycelia grew in WGM medium. (**D**) The aged As2796Y mycelia grew in WGM medium.

The morphological characteristics of As2796 and As2796Y mycelia in PDA and WGM medium were observed after vegetative growth. Both As2796 and As2796Y mycelia exhibited distinct features. The As2796 mycelial colonies exhibited dense white aerial mycelia characterized by a cotton-like texture (Figure 1A). The As2796 mycelial colonies displayed a limited presence of aerial mycelia, with the mycelia exhibiting a brown-yellow hue (Figure 1B). The mycelia of As2796 appeared thick and robust, intertwining densely to form compact networks within the colony. In contrast, the mycelia of As2796Y seemed thinner and less intertwined, resulting in looser networks throughout its colony. The mycelia cultivated in the WGM medium exhibited similar characteristics to those grown in the PDA medium, whereas the aging strain As2796Y produced a significant amount of yellow water on the surface of the mycelia (Figure 1D). Taken together, these results confirmed that As2796Y exhibited the typical characteristics of an aged strain.

### 2.2. Overview of the Transcriptomic Sequencing and Assembly

In this study, a total of six samples were subjected to sequencing, and six corresponding cDNA libraries were constructed, yielding over 237 million raw reads, with a total raw data volume of 35.60 GB (Table S2). After the removal of low-quality reads, 235 million high-quality clean reads were retained. The Q30 base percentage exceeded 92%, indicating a high level of accuracy in the RNA-Seq analysis. Over 85% of these clean reads were successfully mapped to the *A. bisporus* genome, with the majority uniquely mapped (Table S3). Additionally, the boxplot representing the distribution of Fragments Per Kilobase of exon per Million mapped fragments (FPKM) for the As2796 and As2796Y samples showed a consistent trend and a concentrated range of values (Figure S1). These statistics confirm that the quality and quantity of the reads generated are adequate for transcriptomic analysis. Furthermore, gene expression estimates, normalized by FPKM values, revealed that 9925 genes were expressed across all As2796 and As2796Y cDNA libraries (Table S4).

### 2.3. DEGs Identification

**Figure 2:**
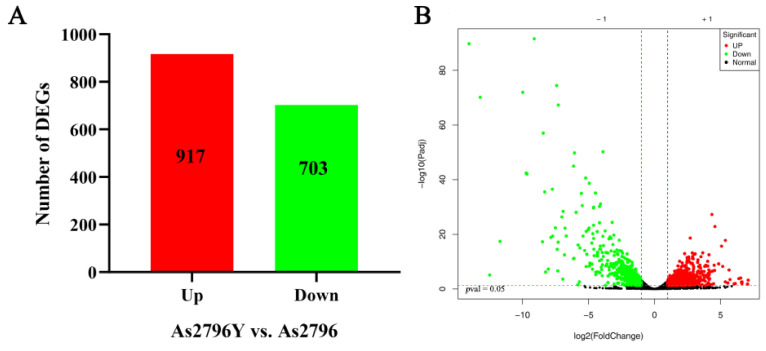
Overview of DEGs analyses for normal As2796 and aged As2796Y spawn. (**A**) Number of DEGs between As2796 and As2796Y spawn. (**B**) Volcano plot of significant DEGs between As2797Y and As2796.

To investigate the variations in transcription levels between normal As2796 spawn and aged As2796Y spawn, DEGs were identified and annotated. This analysis was conducted by comparing the normalized read counts (FPKM values) between As2796Y and As2796. By comparing the normalized read counts (FPKM values) between As2796Y and As2796, a total of 1620 DEGs were identified in the As2796Y group, with 917 genes showing up-regulation and 703 genes displaying down-regulation compared to the As2796 group (Figure 2, Table S5). This significant difference in gene expression patterns was visually represented through a heatmap and sample cluster diagram, highlighting how the aging of spawn significantly impacted the gene expression pattern of *A. bisporus* (Figure S2). Interestingly, upon closer examination, it was observed that many genes in the mycelia of As2796Y exhibited a substantial decrease in expression after aging. These results suggest that these specific genes may play crucial roles in mediating or being involved in the aging process of mycelia.

### 2.4. Functional Annotation of DEGs

**Figure 3:**
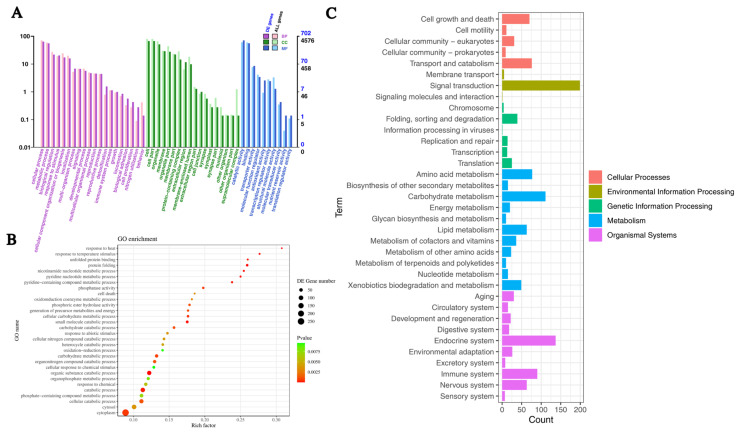
Functional analyses of transcripts within transcriptome. (**A**) Boxplot of GO enrichment analysis of DEGs enriched in BP, CC, and MF. (**B**) Top 30 GO enrichment analysis of down-regulated DEGs. (**C**) KEGG enrichment analysis of DEGs.

In order to gain insights into the functions of crucial DEGs during the aging process of *A. bisporus*, GO enrichment analysis was conducted. The GO enrichment analysis revealed that out of 1620 DEGs in the As2796Y vs. As2796 comparison, 48 terms were enriched, comprising 20 biological processes (BPs), 18 cellular components (CCs), and 10 molecular functions (MFs) (Figure 3A). Among the BPs, significant DEGs were mostly enriched in cellular process (GO:0009987), metabolic process (GO:0008152), biological regulation (GO:0065007), response to stimulus (GO:0050896), cellular component organization or biogenesis (GO:0071840), localization (GO:0051179), and signaling (GO:023052) (Table S6). Among the CCs, significant DEGs were mostly enriched in cell (GO:0005623), cell part (GO:0044464), organelle (GO:0043226), membrane (GO:0016020), organelle part (GO:0044422), and membrane part (GO:0044425) (Table S6). Among the MFs, significant DEGs were mostly enriched in catalytic activity (GO:0003824), binding (GO:0005488), and transporter activity (GO:0005215) (Table S6). The down-regulated expression of enriched DEGs showed similar results to the entire GO enrichment analysis (Figure 3B and Figure S3), suggesting that these down-regulated genes may play a crucial role in the aging process of *A. bisporus* spawn.

In the category of BPs, clusters enriched with significantly down-regulated DEGs primarily focused on processes, such as the organic substance catabolic process, catabolic process, small molecule catabolic process, protein folding, nucleoside diphosphate phosphorylation, ATP generation from ADP, glycolytic process, glucose catabolic process, and nicotinamide nucleotide metabolic process (Figure S4). The findings suggest a decline in the metabolic capacity of carbohydrates within the mycelial cells of aging strains, leading to impaired energy anabolism and reduced ability for ATP synthesis. Concurrently, there is a decrease in cellular protein folding activity. Furthermore, aged As2796 exhibited a significant down-regulation of numerous DEGs associated with temperature stimulus and heat response, suggesting a diminished capability to withstand adverse environmental conditions.

In the category of CCs, clusters enriched with significantly down-regulated DEGs primarily focused on processes, such as the cytoplasm, cytosol, laser plasma membrane, vacuole–mitochondrion membrane contact site, UDP forming (Figure S5). The down-regulated genes observed in As2796Y mycelial cells during aging suggest potential structural damage.

In the category of MFs, clusters enriched with significantly down-regulated DEGs primarily focused on processes, such as the phosphoric ester hydrolase activity, phosphatase activity, unfolded protein binding, heat shock protein binding, protein tyrosine phosphatase activity, chaperone binding, oxidoreductase activity, peptide disulfide oxidoreductase activity, ATPase regulator activity, chaperone binding, superoxide dismutase (SOD) activity (Figure S6). The down-regulation of DEGs in these key molecular functions signals a profound impact on both cellular and mitochondrial operations. The combination of impaired signal transduction, compromised stress responses, and weakened energy metabolism highlights the potential for significant physiological disturbances.

The DEGs were classified to further elucidate their regulatory role, and the enrichment of significant DEGs was analyzed using the KEGG database pathways. The 1620 DEGs were mainly classified into cellular processes, environmental information processing, genetic information processing, amino acid metabolism, and energy metabolism (Figure 3C). The cellular processes exhibited significant enrichment in pathways related to cell cycle, autophagy, lysosome, meiosis, and peroxisome (Table S7). The environmental information processing involves the enrichment of various signal transduction categories and membrane categories, including the sphingolipid signaling pathway, mTOR signaling pathway, MAPK signaling pathway, PI3K-Akt signaling pathway, cAMP signaling pathway, FoxO signaling pathway, and ABC transporters (Table S7). The most significantly enriched pathways in genetic information processing include protein folding, sorting and degradation, as well as transcription processes such as protein processing in the endoplasmic reticulum, spliceosome activity, and RNA degradation (Table S7). The most significantly enriched metabolic categories included carbohydrate metabolism, amino acid metabolism, energy metabolism, lipid metabolism, nucleotide metabolism, xenobiotics biodegradation, and metabolism. Specifically, these encompassed glycine, serine, and threonine metabolism; tyrosine metabolism; pentose and glucuronate interconversions; fructose and mannose metabolism; glycolysis/gluconeogenesis; starch and sucrose metabolism; amino sugar and nucleotide sugar metabolism; pyruvate metabolism; citrate cycle (TCA cycle). Additionally, the oxidative phosphorylation (OXPHOS) pathway associated with energy production exhibited significant enrichment (Table S7). The lipid metabolism pathways were primarily composed of glycerophospholipid metabolism, glycerolipid metabolism, sphingolipid metabolism, and fatty acid degradation, which were significantly enriched. Additionally, numerous metabolic pathways were also enriched in organismal systems, such as the longevity regulating pathway in the aging category and growth hormone synthesis, secretion and action in the endocrine system category.

### 2.5. DEGs Related to Mitochondrial Function

In this study, a series of DEGs closely related to mitochondrial function were identified through comparative transcriptome analysis of normal and aged *A. bisporus* mycelia. The comparative analysis revealed a significant association between 101 DEGs and mitochondrial functions, primarily encompassing vital biological pathways, such as the mitochondrial electron transport chain (ETC), TCA cycle, fatty acid β-oxidation, ATP synthesis, reactive oxygen species (ROS) scavenging, amino acid degradation, and maintenance of mitochondrial membranes (Table S8). These pathways are essential for maintaining proper mitochondrial function and overall cellular health. The expression patterns of these genes suggest a potential pivotal role of mitochondrial dysfunction in the aging process of mushroom mycelia culture.

#### 2.5.1. DEGs Related to ETC

**Figure 4:**
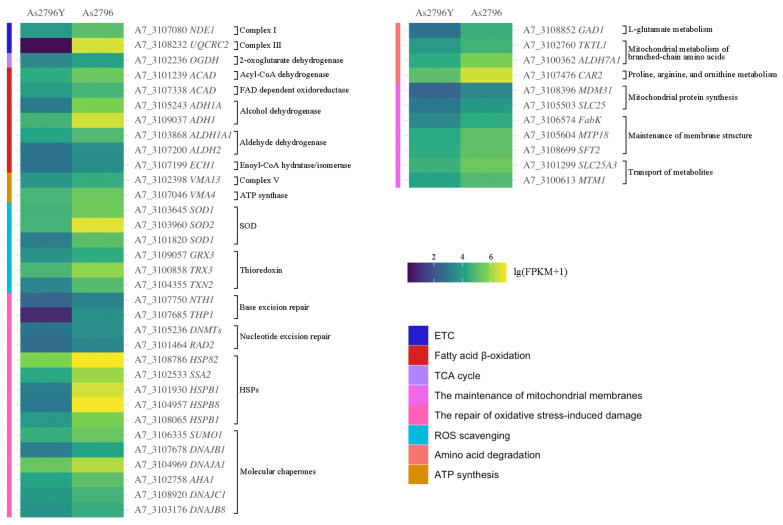
Heatmap of main DEGs involved in mitochondrial function. The different colors indicate the expression level changes between As2796Y and As2796 with lg(FPKM + 1).

Among the DEGs identified in our analysis, we found several key genes that are closely associated with the ETC. Specifically, we observed significant down-regulation of genes encoding components of NADH dehydrogenase (complex I), and ubiquinol-cytochrome c reductase complex (complex III) when comparing As2796Y to As2796. The ETC is a crucial metabolic pathway responsible for generating energy in cells. Complex I, also known as NADH dehydrogenase or respiratory chain complex I, plays a vital role in transferring electrons from NADH to ubiquinone during OXPHOS. The proton gradient generated by complex III (and other ETC complexes) drives ATP synthase (complex V) to synthesize ATP. Complex III is also a site of ROS production. When electron transfer is obstructed, electrons may leak to oxygen during the electron transport process, forming superoxide radicals (·O^2−^). In this study, we observed a reduction in the expression levels of subunits involved in the assembly of complex I and complex III. Specifically, genes *A7_3107080* and *A7_3108232* encode subunits essential for the proper functioning and stability of complex I and complex III (Figure 4). The down-regulation of these genes suggests potential impairment or dysfunction within the ETC pathway, which could lead to decreased ATP synthesis and increased ROS production.

#### 2.5.2. DEGs Related to TCA Cycle

The TCA cycle involves a series of chemical reactions that convert carbohydrates, fats, and proteins into carbon dioxide and energy-rich molecules such as NADH and FADH2. One specific gene involved in the TCA cycle is *OGDH* (*A7_3102236*), which encodes an enzyme called 2-oxoglutarate dehydrogenase (Figure 4). This enzyme catalyzes an important step in the TCA cycle by converting 2-oxoglutarate into succinyl-CoA. Succinyl-CoA then enters further reactions to generate ATP. In this particular case, with aged As2796Y, it was found that there was significant down-regulation of OGDH gene expression. This suggests that there may be alterations in the functioning of the TCA cycle in aged As2796Y individuals.

#### 2.5.3. DEGs Related to Fatty Acid β-Oxidation

The analysis revealed significant changes in the expression of genes involved in fatty acid β-oxidation, a crucial metabolic pathway for energy production. Specifically, the down-regulation of two key genes, encoding acyl-CoA dehydrogenase (*A7_3101239*) and FAD-dependent oxidoreductase (*A7_3107338*), in aged As2796Y spawn suggests potential impairment in the catabolism of fatty acids and subsequent energy production. Furthermore, several other genes responsible for synthesizing alcohol dehydrogenase (ADH, *A7_3105243*, *A7_3109037*) and aldehyde dehydrogenase (ALDH, *A7_3103868*, *A7_3107200*) were also significantly down-regulated. ADH enzymes play an essential role in converting alcohols into their corresponding aldehydes or ketones, while ALDH enzymes are responsible for further oxidizing these compounds into carboxylic acids. The decreased expression of these genes indicates a possible disruption in alcohol metabolism and detoxification processes within aging As2796Y mycelia. The expression of *ECH1* (*A7_3107199*), an important gene involved in the unsaturated fatty acid β-oxidation, also showed a significant decrease trend in As2796Y spawn (Figure 4).

#### 2.5.4. DEGs Related to ATP Synthesis

Similarly, we observed a decrease in the expression levels of genes encoding subunits of complex V (*A7_3102398*) or ATP synthase (*A7_3107046*) (Figure 4). Complex V is responsible for synthesizing ATP by utilizing the proton gradient generated during electron transport through complexes I-IV. The reduced expression levels of these two genes suggest a potential disruption in ATP synthesis efficiency, which could impair energy-dependent processes essential for cell maintenance and growth.

#### 2.5.5. DEGs Related to ROS Scavenging

Genes involved in the scavenging of ROS exhibited differential expression, with several crucial antioxidant genes encoding superoxide dismutase (SOD, *A7_3103645*, *A7_3103960*, *A7_3101820*) and thioredoxin (*A7_3109057*, *A7_3100858*, *A7_3104355*) being down-regulated in As2796Y (Figure 4). This reduction in antioxidant capacity likely exacerbates oxidative stress within aging mycelia, contributing to cellular damage and senescence. The impaired ability to efficiently eliminate ROS can result in oxidative damage to proteins, lipids, and DNA molecules, thereby accelerating the aging process and potentially leading to cell death.

#### 2.5.6. DGEs Associated with the Repair of Oxidative Stress-Induced Damage

Mitochondria possess various repair mechanisms to cope with oxidative stress-induced damage, including mitochondrial DNA repair and protein repair and degradation. In this study, the expression of numerous genes involved in the synthesis of mitochondrial DNA repair enzymes was significantly diminished in As2796Y, such as base excision repair coding genes (*A7_3107750*, *A7_3107685*) and nucleotide excision repair coding genes (*A7_3105236*, *A7_3101464*) (Figure 4). Furthermore, a notable down-regulation trend was observed in many genes associated with protein repair and degradation processes, such as heat shock protein (HSP) coding genes (*A7_3108786*, *A7_3102533*, *A7_3101930*, *A7_3104957*, *A7_3108065*) and molecular chaperone coding genes (*A7_3106335*, *A7_3107678*, *A7_3104969*, *A7_3102758*, *A7_3108920*, *A7_3103176*) (Figure 4).

#### 2.5.7. DEGs Related to Amino Acid Degradation

The mitochondria play a crucial role in various amino acid metabolic processes, which are indispensable for maintaining mitochondrial function. These processes encompass the deamination of amino acids and the subsequent metabolism of their carbon skeletons. In this study, the expression of gene (*A7_3108852*) involved in the metabolic pathway of L-glutamate to produce succinate, which subsequently enters the TCA cycle, was found to be significantly down-regulated in the aged spawn (Figure 4). Moreover, there was a significant down-regulation observed in the expression of multiple genes (*A7_3102760*, *A7_3100362*) responsible for encoding enzymes involved in the mitochondrial metabolism of branched-chain amino acids (leucine, isoleucine, and valine). The metabolism of proline, arginine, and ornithine also takes place in mitochondria simultaneously, with these amino acids playing crucial roles in the urea cycle and TCA cycle. However, a significant down-regulation of associated gene (*A7_3107476*) was observed in aged As2796Y mycelium.

#### 2.5.8. DEGs Related to the Maintenance of Mitochondrial Membranes

The maintenance of mitochondrial membrane integrity is essential for optimal mitochondrial function. In this study, numerous genes associated with mitochondrial protein synthesis (*A7_3108396*, *A7_3105503*), maintenance of membrane structure (*A7_3106574*, *A7_3105604*, *A7_3108699*), and transport of metabolites (*A7_3101299*, *A7_3100613*) showed significantly down-regulated expression (Figure 4). These genes play a crucial role in preserving normal mitochondrial function through diverse mechanisms. Suppression of the expression of these genes may result in impaired mitochondrial function, subsequently impacting cellular energy metabolism and overall health.

#### 2.5.9. Other Crucial DEGs Implicated in the Maintenance of Mitochondrial Function

**Figure 5:**
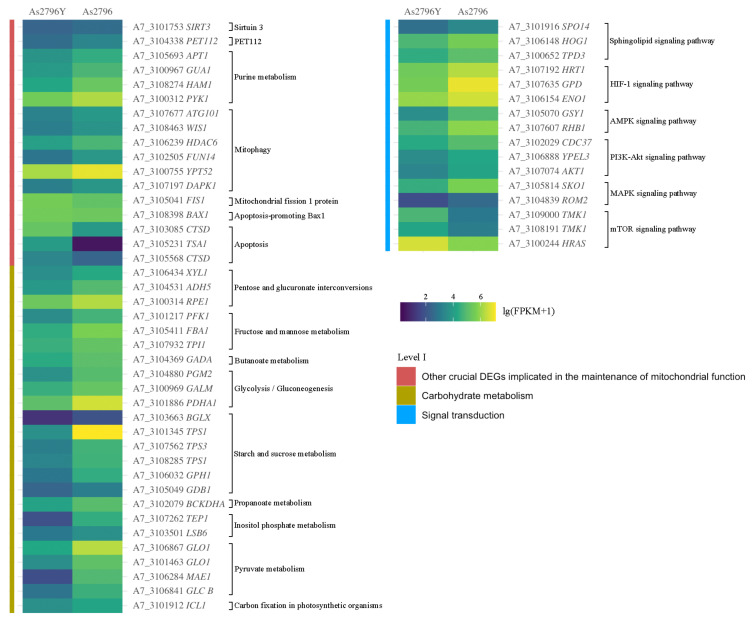
Heatmap of other DEGs involved in mitochondrial function. The different colors indicate the expression level changes between As2796Y and As2796 with lg (FPKM + 1).

The identification of additional DEGs also revealed their crucial involvement in the maintenance of mitochondrial function. These genes are involved in various cellular processes, including energy production, oxidative stress response, and apoptosis regulation. One such gene implicated in maintaining mitochondrial function was *SIRT3* (*A7_3101753*), which showed significant down-regulation in the As2796Y vs. As2796 comparison. This gene encodes a protein that belongs to the sirtuin family of proteins, known for their involvement in regulating cellular metabolism and aging. The gene encoding PET112 (*A7_3104338*) exhibited significant down-regulation in aged As2796Y spawn (Figure 5). The PET112 protein plays a crucial role in mitochondrial tRNA synthesis and modification, thereby ensuring the accuracy and efficiency of protein synthesis. Additionally, it indirectly impacts mitochondrial energy metabolism and overall function, which are essential for cellular energy production and metabolic homeostasis. The expression of several genes (*A7_3105693*, *A7_3100967*, *A7_3108274*, *A7_3100312*) involved in purine metabolism was down-regulated, resulting in a decrease in the activity of key enzymes within the purine synthesis pathway, including IMP, GMP, and AMP synthesis and conversion (Figure 5). Consequently, this leads to a reduction in the synthesis of crucial nucleotides such as ATP and GTP, directly impacting mitochondrial energy metabolism and nucleic acid synthesis. The expression of numerous genes related to mitophagy (*A7_3107677*, *A7_3108463*, *A7_3106239*, *A7_3102505*, *A7_3100755*, *A7_3107197*) was significantly down-regulated in As2796Y spawn (Figure 5). On the contrary, the up-regulation of certain genes can also result in impaired mitochondrial function. For instance, the overexpression of *FIS1* (*A7_3105041*) in As2697Y mycelium could result in excessive mitochondrial division, thereby compromising the stability of the mitochondrial network and leading to mitochondrial dysfunction. Additionally, there was a significant increase in the expression of the proapoptotic gene *Bax1* (*A7_3108398*) in aged As2796Y mycelia. Furthermore, numerous genes (*A7_3103085*, *A7_3105231*, *A7_3105568*) involved in the process of apoptosis were significantly up-regulated in aged As2796Y spawn (Figure 5). The up-regulation of these genes suggests an increased susceptibility to apoptosis or programmed cell death in these cells. When analyzing carbohydrate metabolism pathways, it becomes evident that several crucial routes significantly impact mitochondrial function. These include reduced substrate availability for energy production, compromised nucleotide synthesis, weakened antioxidant defenses, and disrupted cell signaling (Figure 5). The analysis of signal transduction reveals that the expression of multiple genes in several crucial signaling pathways associated with mitochondrial function was also down-regulated in As2796Y spawn (Figure 5). This suggests a potential disruption or impairment in important cellular signaling mechanisms that regulate various physiological processes, including metabolism and growth.

### 2.6. Validation of RNA-Seq Data by qRT-PCR

**Figure 6:**
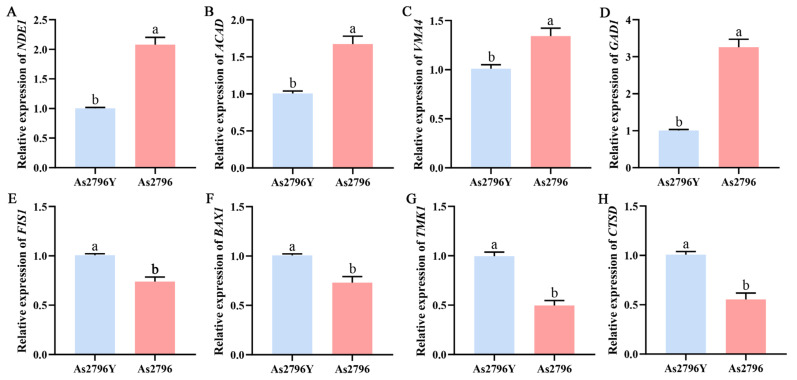
Validation of RNA-Seq results with qRT-PCR. (**A**–**H**) Relative expression of eight genes (*NED1*, *ACAD*, *VMA4*, *GAD1*, *FIS1*, *BAX1*, *TMK1*, and *CTSD*) in As2796 and As2796Y. Data are represented by mean ± SD. Different letters indicate significant differences between the column (*p* ≤ 0.05 according to Duncan’s range test).

To validate the reliability of the DEGs assembled by the RNA-Seq, eight genes correlated with mitochondrial function between the normal and aged *A. bisporus* spawn were selected for qRT-PCR analysis. Their expression profiles were verified to confirm the accuracy of transcriptome analysis data (Figure 6). The results demonstrated a consistent gene expression level between qRT-PCR analysis and RNA-Seq analysis, thereby confirming the reliability and stability of RNA-Seq data.

## 3. Discussion

**Figure 7:**
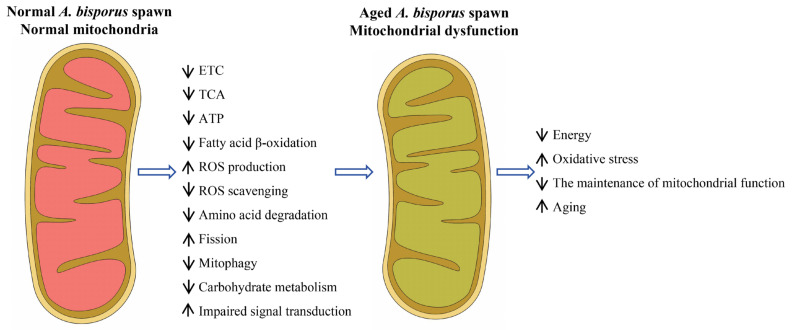
Schematic diagram of a model of mitochondrial dysfunction in *A. bisporus* spawn aging.

The process of aging is a natural, gradual, and inevitable phenomenon that encompasses a series of molecular, cellular, and tissue-level changes [13]. The aging of *A. bisporus* spawn is a critical issue that significantly impacts its production efficiency and product quality. The present study provides valuable insights into understanding how age-related changes affect gene expression patterns within *A. bisporus* spawn samples. By identifying DEGs associated with aging, this study contributes to our knowledge about potential molecular mechanisms underlying mitochondrial dysfunction and *A. bisporus* spawn aging (Figure 7).

To address the issue of *A. bisporus* aging, extensive research has been conducted by scientists and producers, who have implemented various approaches, including optimizing culture conditions, regularly updating strains, enhancing management practices, and employing cryopreservation technology to delay the aging process [14]. However, these methods cannot be fundamentally effective in preventing or reducing the occurrence of *A. bisporus* aging. The underlying reason for this is the limited understanding of the internal molecular mechanisms governing the aging process in edible fungi spawn. Mitochondria play a pivotal role in cellular energy metabolism, and their dysfunction is a hallmark of aging [15]. The functional enrichment analysis comparing As2796Y vs. As2796 revealed significant differential expression of multiple genes involved in key regulatory pathways governing mitochondrial function and genetic information processing (Figure 3). In this study, several down-regulated genes involved in the mitochondrial ETC, TCA cycle, and ATP synthesis in aged *A. bisporus* spawn suggest a direct link between mitochondrial impairment and spawn aging. Notably, the down-regulation of ETC components, including NADH dehydrogenase (complex I) and ubiquinol-cytochrome c reductase (complex III), indicates impaired electron transport and ATP synthesis (Figure 4). These complexes play a crucial role in ATP production, and their impairment leads to reduced cellular energy levels, increased production of ROS, and oxidative damage [16]. The decrease in mitochondrial potential and ATP output, which is closely associated with the reduced expression of these genes, consequently affects the growth rate and morphological characteristics of mycelia. Our previous studies also revealed that aged As2796Y strains exhibit decreased levels of various intermediates in the TCA cycle and substances involved in maintaining cellular stability [2]. Additionally, the down-regulated expression of several ATP synthase subunits involved in ATP synthesis in aged As2796Y mycelia further supports the decline in energy metabolism during aging (Figure 4). The down-regulation of these genes in aged spawn suggests impaired mitochondrial respiration and energy production, which could contribute to the aging process.

The observed down-regulation of genes involved in fatty acid β-oxidation, such as acyl-CoA dehydrogenase and FAD-dependent oxidoreductase, suggests impaired catabolism of fatty acids (Figure 4). The reduction in β-oxidation may lead to the intracellular accumulation of fatty acids, which can have toxic effects and contribute to further metabolic disturbances. Impaired β-oxidation restricts the breakdown of fatty acids for energy production, thereby reducing energy availability and increasing oxidative stress due to incomplete fatty acid metabolism [17]. Similarly, impaired amino acid degradation in aged As2796Y spawn can also lead to the accumulation of toxic metabolic intermediates, further contributing to cellular dysfunction and aging [18]. This highlights the importance of maintaining amino acid metabolism for mitochondrial and cellular health.

Carbohydrate metabolism pathways are crucial for providing substrates for the TCA cycle and ETC. The mitochondria heavily depend on the products of glycolysis and the TCA cycle for efficient oxidative phosphorylation and ATP production [19]. The genes *XYL1*, *ADH5*, and *RPE1* involved in the pentose and glucuronate interconversions pathway, as well as *ENO1* and *PGM2* in the glycolysis/gluconeogenesis pathway, exhibit significant down-regulation (Figure 5). This disruption in metabolic pathways will contribute to decreased ATP synthesis and energy metabolism disorders [20]. Moreover, the interconversion of pentose and glucuronate is crucial for nucleotide synthesis. Impairment in this pathway will result in a decrease in nucleotide synthesis, thereby impacting the synthesis of mitochondrial DNA and RNA. Consequently, it compromises the mitochondria’s ability to replicate and transcribe, ultimately leading to mitochondrial dysfunction [21]. The observed down-regulated genes in fructose and mannose metabolism (*PFK1*, *FBA1*), butanoate metabolism (*GADA*), and glycolysis (*PDHA1*) directly impair the cell’s ability to efficiently convert glucose into pyruvate and subsequently enter the TCA cycle. The reduced enzymatic activity in glycolysis could lead to a significant decrease in pyruvate production, which is a crucial substrate for mitochondrial respiration. Furthermore, the observed down-regulation of genes involved in starch and sucrose metabolism indicates a diminished capacity for glycogen synthesis and breakdown. A reduced supply of substrates due to down-regulated carbohydrate metabolism genes results in impaired mitochondrial function, leading to inadequate ATP generation and the potential accumulation of ROS [22]. These metabolic impairments closely correlate with mitochondrial dysfunction. In summary, various aspects of carbohydrate metabolism are closely related to mitochondrial function, and any abnormalities can significantly impact mitochondrial energy production and cellular metabolism.

The accumulation of ROS is a major consequence of mitochondrial dysfunction, and the ability to scavenge these ROS is crucial for maintaining cellular health [23]. The differential expression of genes involved in ROS scavenging and oxidative stress repair highlights significant impacts on cellular aging and mitochondrial function [15]. In this study, we observed a marked down-regulation of several key antioxidant genes encoding SOD and thioredoxin in As2796Y (Figure 4). This down-regulation likely exacerbates oxidative stress within aging mycelia by reducing the overall antioxidant capacity, which plays a critical role in neutralizing ROS. Insufficient elimination of ROS can result in oxidative damage to proteins, lipids, and DNA molecules, accelerating cellular senescence and potentially leading to cell death [24]. Additionally, the impaired expression of genes associated with mitochondrial repair mechanisms and protein repair and degradation in aged As2796Y spawn exacerbates cellular oxidative damage, compromising the ability to repair DNA damage and reducing the capacity for maintaining protein homeostasis caused by oxidative stress within mitochondria (Figure 4). This impairment can lead to the accumulation of mitochondrial DNA mutations and damaged proteins, further worsening mitochondrial function and aging.

The mitochondria possess a sophisticated quality control system that restricts mitochondrial damage to ensure the integrity and functionality of these organelles. In this study, several genes associated with the maintenance of mitochondrial membrane integrity showed significant down-regulation in aged As2796Y (Figure 4). Previous studies have demonstrated that the down-regulation of genes, such as MDM31 involved in yeast mitochondrial distribution and morphology, and *SLC25A8*, a mitochondrial carrier, can significantly impact mitochondrial protein synthesis [25,26]. Additionally, *FabK*, which encodes enoyl-(acyl carrier protein) reductase, an enzyme involved in fatty acid biosynthesis, is necessary for mitochondrial membrane formation [27]. The proteins encoded by these genes play a crucial role in maintaining the structural components of the mitochondrial membrane. Down-regulation can result in defective protein synthesis, leading to compromised membrane structure and impaired mitochondrial function. Similarly, *SIRT3* specifically targets mitochondria and plays a crucial role in modulating various aspects of mitochondrial physiology, including ATP production, detoxification of reactive oxygen species, and fatty acid oxidation [28]. In this study, the expression of *SIRT3* in aged As2796Y spawn was significantly down-regulated (Figure 5). A previous study found that the down-regulation of *SIRT3* leads to increased acetylation levels of mitochondrial proteins, impairing their activity and stability. Consequently, this results in the accumulation of ROS, compromised metabolic function, disrupted mitochondrial dynamics balance, and enhanced apoptosis, ultimately leading to cellular damage caused by mitochondrial dysfunction [17]. Moreover, the down-regulation of PET112, a nuclear gene that encodes a protein involved in the mitochondrial translation machinery, will further exacerbate mitochondrial dysfunction in the aged As2796Y mycelium [29]. The aforementioned findings further support the innovative discovery that the aged As2796Y mycelium exhibits impaired functionality in maintaining its own mitochondrial function.

The intricate regulatory network that maintains a balance between the generation of new mitochondria and the removal of damaged ones forms the foundation for aging and longevity [30]. The maintenance of mitochondrial and cellular homeostasis necessitates precise regulation and coordination between the generation of new mitochondria and the removal of damaged ones. The selective degradation of dysfunctional or impaired mitochondria is achieved through a specialized autophagy process called mitophagy, while the synthesis of fresh mitochondria occurs via mitochondrial biogenesis. The significant down-regulation of mitophagy-related genes in aged As2796Y mycelia further indicates impaired mitochondrial clearance mechanisms, potentially resulting in the accumulation of damaged mitochondria and cellular aging [31]. Conversely, the overexpression of certain genes also results in impaired mitochondrial function. For instance, the overexpression of *FIS1* in As2796Y could lead to excessive mitochondrial division, compromising the stability of the mitochondrial network and causing mitochondrial dysfunction. The significant up-regulation of the pro-apoptotic gene Bax1 in aged As2796Y mycelia further suggests an increased susceptibility to apoptosis or programmed cell death in these cells [32]. Additionally, a significant up-regulation of numerous genes associated with the apoptotic process was observed in aged As2796Y mycelia, suggesting an elevated susceptibility to programmed cell death in these cells [33]. This pattern of gene expression may consequently impair cell survival capacity and subsequently impact the growth and reproductive potential of the mycelium. The analysis of signal transduction revealed that the expression of multiple genes in crucial signaling pathways associated with mitochondrial function was down-regulated in As2796Y mycelia, potentially disrupting important cellular signaling mechanisms regulating various physiological processes, including metabolism and growth [33]. This impairment may be a significant reason for dysfunctional cellular functions during aging. Therefore, the persistent mitochondrial dysfunction and impaired repair mechanisms in *A. bisporus* mycelia during the cultivation process ultimately result in spawn aging. Furthermore, recent studies on the mitochondrial genome of *A. bisporus* have significantly advanced our understanding of its structure, gene organization, and functional attributes, particularly those related to energy metabolism and aging, including the subunits of the electron transport chain and the ATP–synthase complex [34]. Building on these findings, our study enhances the current understanding of mitochondrial genome research by providing a dynamic perspective on the role of transcriptomic changes in the aging process. Future research could further advance this field by integrating genomic and transcriptomic datasets to better clarify the functional implications of mitochondrial alterations in *A. bisporus*.

## 4. Materials and Methods

### 4.1. Strains, Media, Growth Conditions, and Sample Collection

*A. bisporus* As2796 (CGMCC0214) and *A. bisporus* As2796Y (CGMCC0214-a) were obtained from China General Microbiological Culture Collection Center. The strain As2796Y is an aged variant of As2796 that was identified during the cultivation process [2]. To observe the morphological characteristics of As2796 and As2796Y mycelia, a mycelial disc (diameter: 5 mm) was inoculated on the center of potato dextrose agar (PDA, Difco-Becton Dickinson, Sparks, MD, USA) at 24 °C for 20 days. The wheat spawn was obtained by inoculating five mycelial discs (5 mm) into a wheat grain medium (WGM, consisting of 92% wheat, 4% gypsum, and 4% light calcium carbonate) with a water content of 47%, in a bag measuring 17 × 35 cm. The mixture was then incubated at a temperature of 24 °C for 20 days. To investigate the differential transcription levels between normal and aged spawn, mycelial samples were collected after 20 days of wheat spawn cultivation. The individual mycelia were analyzed in triplicate for As2796 and As2796Y wheat spawn, rapidly frozen in liquid nitrogen, and subsequently stored at –80 °C until RNA extraction.

### 4.2. RNA Extraction

Total RNA was extracted from the tissue using the RNeasy Plant Mini Kit (Qiagen, Inc, Valencia, CA, USA) according to the manufacturer’s protocol. Then, RNA quality was determined using Agilent 2100 Bioanalyser (Agilent Technologies, Palo Alto, CA, USA) and quantified using the ND-3300 (NanoDrop Technologies, Inc., Wilmington, DE, USA). Only high-quality RNA sample (OD260/280 = 1.8~2.2, OD260/230 ≥ 2.0, RIN ≥ 6.5, 28S:18S ≥ 1.0, >1 μg) was used to construct sequencing library.

### 4.3. cDNA Library Preparation, and Illumina Sequencing

A total of 1 μg RNA sample was used for the RNA-Seq transcriptome library using the TruSeqTM RNA sample preparation kit from Illumina (San Diego, CA, USA). Briefly, messenger RNA was isolated through polyA selection using oligo (dT) beads and then fragmented using a fragmentation buffer. Subsequently, double-stranded cDNA was synthesized using the SuperScript double-stranded cDNA synthesis kit (Invitrogen, Carlsbad, CA, USA) with random hexamer primers (Illumina). The synthesized cDNA underwent end-repair, phosphorylation, and ‘A’ base addition following Illumina’s library construction protocol. Libraries were size selected for cDNA target fragments of 300 bp on a 2% Low-Range Ultra Agarose gel and then PCR amplified for 15 cycles using Phusion DNA polymerase (NEB, Beijing, China). After quantification by TBS380, the paired-end RNA-Seq sequencing library was sequenced on the Illumina HiSeq xten/NovaSeq 6000 sequencer (Illumina, San Diego, CA, USA) with a read length of 2 × 150 bp. Three independent sequencing libraries were prepared for each treatment. The raw sequence data were submitted to the National Center for Biotechnology Information (NCBI) Sequence Read Archive (SRA), assigned accession number SUB14538921. The raw paired-end reads were subjected to trimming and quality control using SeqPrep (V1.2, https://github.com/jstjohn/SeqPrep, accessed on 19 March 2023) and Sickle (V1.33, https://github.com/najoshi/sickle, accessed on 19 March 2023) with default parameters. Subsequently, the clean reads were individually aligned to the reference *A. bisporus* genome (PRJNA635555) in orientation mode using HISAT2 software (V2.2.1, http://ccb.jhu.edu/software/hisat2/index.shtml, accessed on 20 March 2023). The mapped reads from each sample were then assembled through a reference-based approach employing StringTie software (V2.2.0, https://ccb.jhu.edu/software/stringtie/index.shtml?t=example, accessed on 22 March 2023).

### 4.4. Differential Expression Analysis and Functional Enrichment

To identify differentially expressed genes (DEGs) between As2796 and As2796Y samples, the expression level of each transcript was quantified using the transcripts per million reads (TPM) method. Gene abundances were calculated using RSEM (V1.3.3, http://deweylab.biostat.wisc.edu/rsem/, accessed on 25 March 2023) [35]. Differential expression analysis was performed using DESeq2/DEGseq/EdgeR with a Q value ≤ 0.05, considering DEGs with |log_2_FC| > 1 and Q value ≤ 0.05 (DESeq2 or EdgeR)/Q value ≤ 0.001 (DEGseq) as significantly differentially expressed genes [36,37,38]. Additionally, functional enrichment analysis including gene ontology (GO) and Kyoto Encyclopedia of Genes and Genomes (KEGG) was conducted to identify significantly enriched DEGs in GO terms and metabolic pathways at a Bonferroni-corrected *p*-value ≤ 0.05 compared to the whole-transcriptome background. GO functional enrichment and KEGG pathway analysis were carried out using Goatools (V1.3.11, https://github.com/tanghaibao/Goatools, accessed on 26 March 2023) and KOBAS (V3.0.3, http://kobas.cbi.pku.edu.cn/home.do, accessed on 26 March 2023) [39].

### 4.5. Quantitative Real-Time PCR

The relative expression levels of the targeted genes in the As2796 and As2796Y samples were validated to confirm the RNA-Seq results. A total of 8 randomly selected genes associated with mitochondrial function were quantified by qRT-PCR. The SYBR Premix Ex Taq II kit from TaKaRa (Dalian, China) was utilized, and the reaction was conducted on the Bio-Rad CFX96 System (Bio-Rad Laboratories, Inc., Hercules, CA, USA). Each sample was conducted with three replicates. The selected gene expression levels were normalized using GADPH as an internal control and determined using the 2^−ΔΔCt^ method. The primers used for qPCR analysis are provided in Supplementary Table S1.

### 4.6. Statistical Analysis

The statistical analyses were conducted using SPSS 20.0 (SPSS Inc., Chicago, IL, USA). The data are presented as the means ± standard deviation (SD) from a minimum of three biological replicates. Statistically significant differences were determined through analysis of variance (ANOVA) and Duncan’s multiple range tests (*p* ≤ 0.05).

## 5. Conclusions

In conclusion, this study represents a comprehensive effort to explore the mechanism underlying spawn aging in *A. bisporus* through transcriptome analysis. A mitochondrial dysfunction model underlying *A. bisporus* spawn aging was proposed. Our findings first provide new insights into the role of mitochondrial dysfunction in the aging process of *A. bisporus* spawn. Overall, this study sheds light on the intricate relationship between mitochondrial function and aging in the *A. bisporus* mycelia culture. The identification of these DEGs associated with key metabolic pathways emphasizes their significance in maintaining optimal cellular functioning during both normal growth and aging processes. Further research can build upon these findings to explore targeted interventions that aim to preserve or enhance mitochondrial function, ultimately improving the longevity and productivity of this commercially important mushroom species.

## Data Availability

All data presented in this study are available within this article.

## Supplementary Materials

The following supporting information can be downloaded at: https://www.mdpi.com/article/10.3390/ijms26020849/s1.
