# Supplementary material for: Comparative Transcriptomic Analysis Reveals New Insights into Spawn Aging in Agaricus bisporus: Mitochondrial Dysfunction
Source: Int J Mol Sci. 2025 Jan 20;26(2):849. doi: 10.3390/ijms26020849 (PMC11766156; doi:10.3390/ijms26020849)
Supplement: Supplementary file 1 [file ijms-26-00849-s001.zip › ijms-3426991-supplementary/Supplementary Figures captions.pdf]

Figure S1: Boxplot of FPKM distribution;
Figure S2: The heatmap of significant DEGs in As2796Y vs As2796 comparison;
Figure S3: GO enrichment analysis of down-regulated DEGs;
Figure S4: Bubble plots of clusters enrichment analysis of down-regulated DEGs among the category of BP;
Figure S5: Bubble plots of clusters enrichment analysis of down-regulated DEGs among the category of CC;
Figure S6: Bubble plots of clusters enrichment analysis of down-regulated DEGs among the category of MF
