# Supplementary material for: Comparative Transcriptomic Analysis Reveals New Insights into Spawn Aging in Agaricus bisporus: Mitochondrial Dysfunction
Source: Int J Mol Sci. 2025 Jan 20;26(2):849. doi: 10.3390/ijms26020849 (PMC11766156; doi:10.3390/ijms26020849)
Supplement: Supplementary file 1 [file ijms-26-00849-s001.zip › ijms-3426991-supplementary/Supplementary Table S1-S3.pdf]

Supplementary Material

Table S1. The list primers for qRT-PCR validation.

Gene ID	Gene name	Description	Primers sequence(5’-3’)
A7_3107080	*NDE1*	NADH dehydrogenase	Forward: TTCGGAATCGTACTCTCGTCG<br>Reverse: TTCACGAGCGACATCACTGTA
A7_3107338	*ACAD*	FAD dependent oxidoreductase	Forward: GTGGACTTCGACCTGCAAGA<br>Reverse: AAACTGACAACGCAAACGCA
A7_3107046	*VMA4*	ATP synthase (E/31 kDa) subunit	Forward: AGACGGGCATGTGTTTCTCC<br>Reverse: AACGCACCCTGTCTTCCAAA
A7_3108852	*GAD1*	Belongs to the group II decarboxylase family	Forward: CGCAAAGCCGTACGTCAATTA<br>Reverse: CCGGGCAATATCCAGCAGAT
A7_3105041	*FIS1*	Mitochondrial fission 1 protein	Forward: TGTCGGCAGCACTGTTACTT<br>Reverse: AGCCCTCTGTCAATGTCGTT
A7_3108398	*BAX1*	Apoptosis-promoting Bax1	Forward: GATGACGGGGATCATTGGCA<br>Reverse: GGCTGGGAGACTGAAAGGAC
A7_3108191	*TMK1*	Mitogen-activated protein kinase	Forward: TGGGGAGCACATGGTTTTGT<br>Reverse: AAGCAGGAGCGAGGTAAGGT
A7_3105568	*CTSD*	Belongs to the peptidase A1 family	Forward: TTGACATTTGGCGGAAAGGC<br>Reverse: CTACCCAGACTGTCCGAAGC
A7_3102351	*18S rbs*	18S ribosomal RNA gene	Forward: CTCGCAAGGTCAGTTCTGGT<br>Reverse: GTCCTACTTCGCGTGCTTCT

Table S2. Summary of raw sequencing data and assembly.

Samples	Raw reads	Raw data (G)	GC Content	Q20 (%)	Q30 (%)
As2796_1	40,605,924	6.09	49.07%	97.06%	92.20%
As2796_2	37,832,672	5.67	49.37%	96.99%	92.05%
As2796_3	40,399,080	6.06	49.11%	97.20%	92.46%
As2796Y_1	37,599,110	5.64	49.04%	96.97%	92.00%
As2796Y_2	39,894,282	5.98	49.11%	97.13%	92.42%
As2796Y_3	41,012,680	6.15	49.17%	96.96%	92.07%

Table S3. Mapped results of clean data.

Samples	Clean reads	Mapped reads (%)	Secondary alignments (%)	Uniquely alignments (%)
As2796_1	40,177,996	35,212,928 (87.64)	6,568,032 (16.35)	28,644,896 (71.29)
As2796_2	37,590,908	32,790,105 (87.23)	2,177,231 (5.79)	30,612,874 (81.44)
As2796_3	40,154,354	35,092,934 (87.4)	3,003,422 (7.48)	32,089,512 (79.92)
As2796Y_1	37,343,568	32,303,906 (86.5)	627,564 (1.68)	31,676,342 (84.82)
As2796Y_2	39,633,052	35,123,200 (88.62)	7,443,672 (18.78)	27,679,528 (69.84)
As2796Y_3	40,719,308	35,122,768 (86.26)	4,117,300 (10.11)	31,005,468 (76.14)
