# Supplementary figures and images for: Comparative Transcriptomic Analysis Reveals New Insights into Spawn Aging in Agaricus bisporus: Mitochondrial Dysfunction
Source: Int J Mol Sci. 2025 Jan 20;26(2):849. doi: 10.3390/ijms26020849 (PMC11766156; doi:10.3390/ijms26020849)

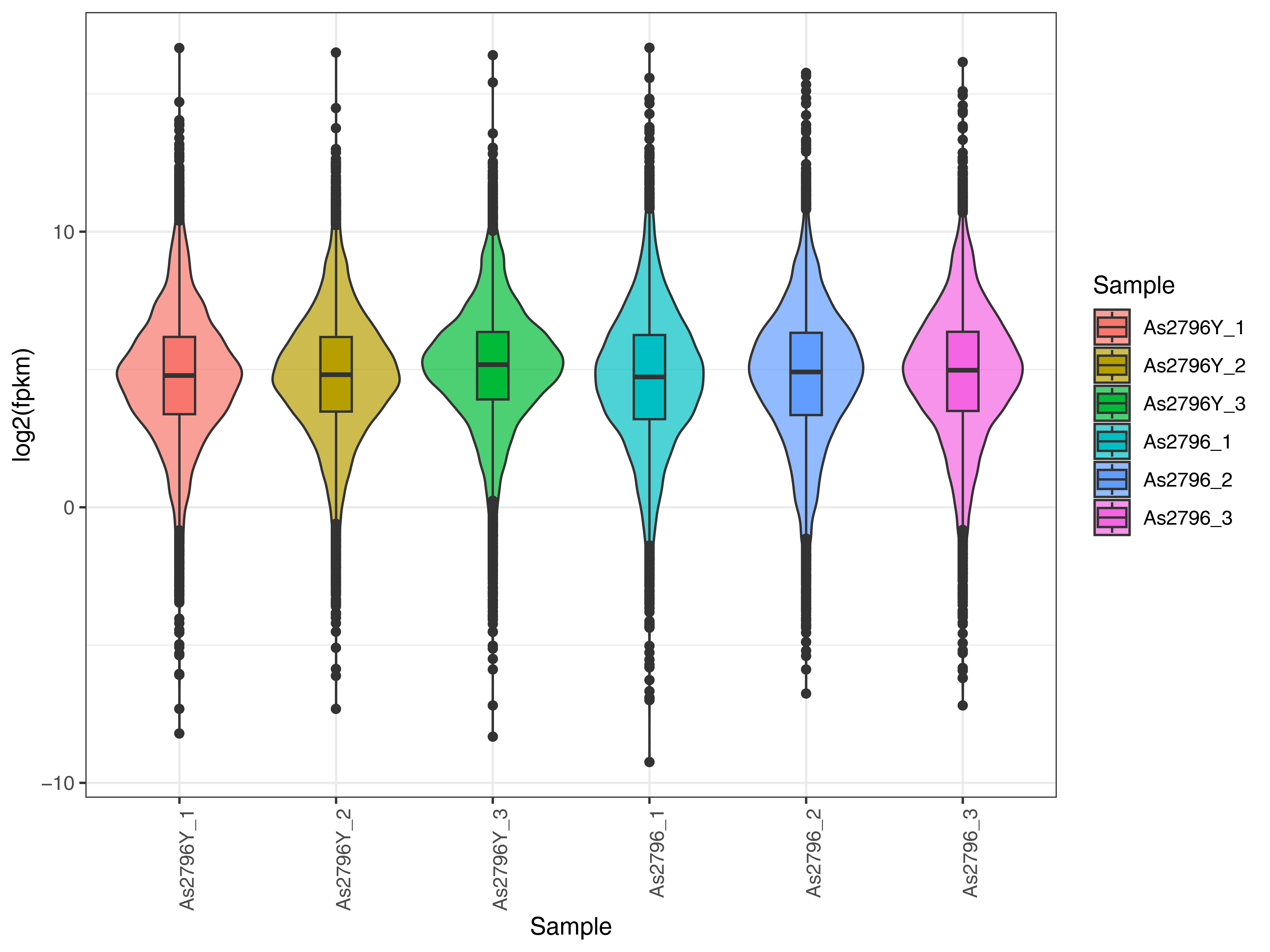

Supplement: Supplementary file 1 [file ijms-26-00849-s001.zip › ijms-3426991-supplementary/Figure. S1.tif]

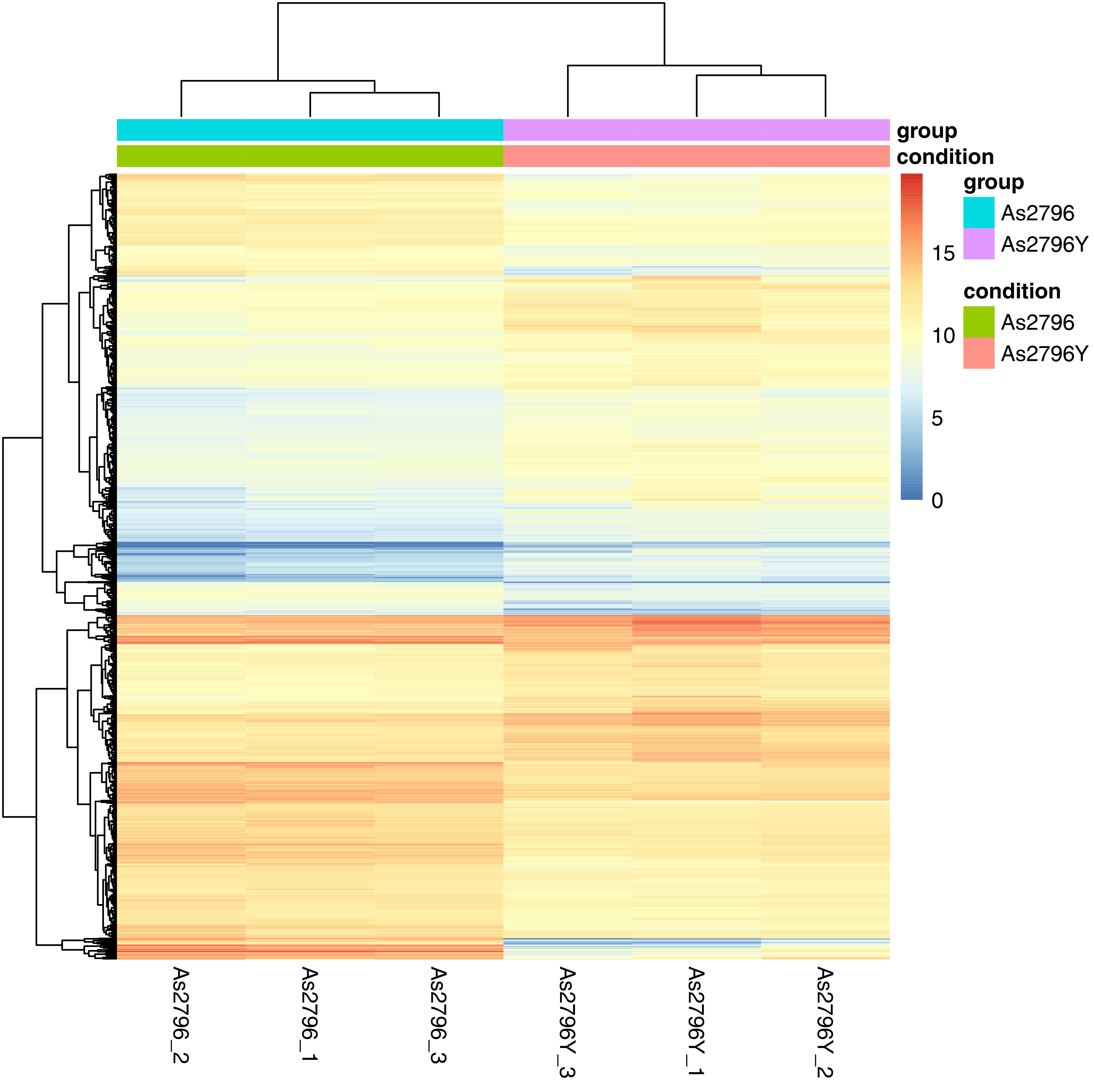

Supplement: Supplementary file 1 [file ijms-26-00849-s001.zip › ijms-3426991-supplementary/Figure. S2.tif]

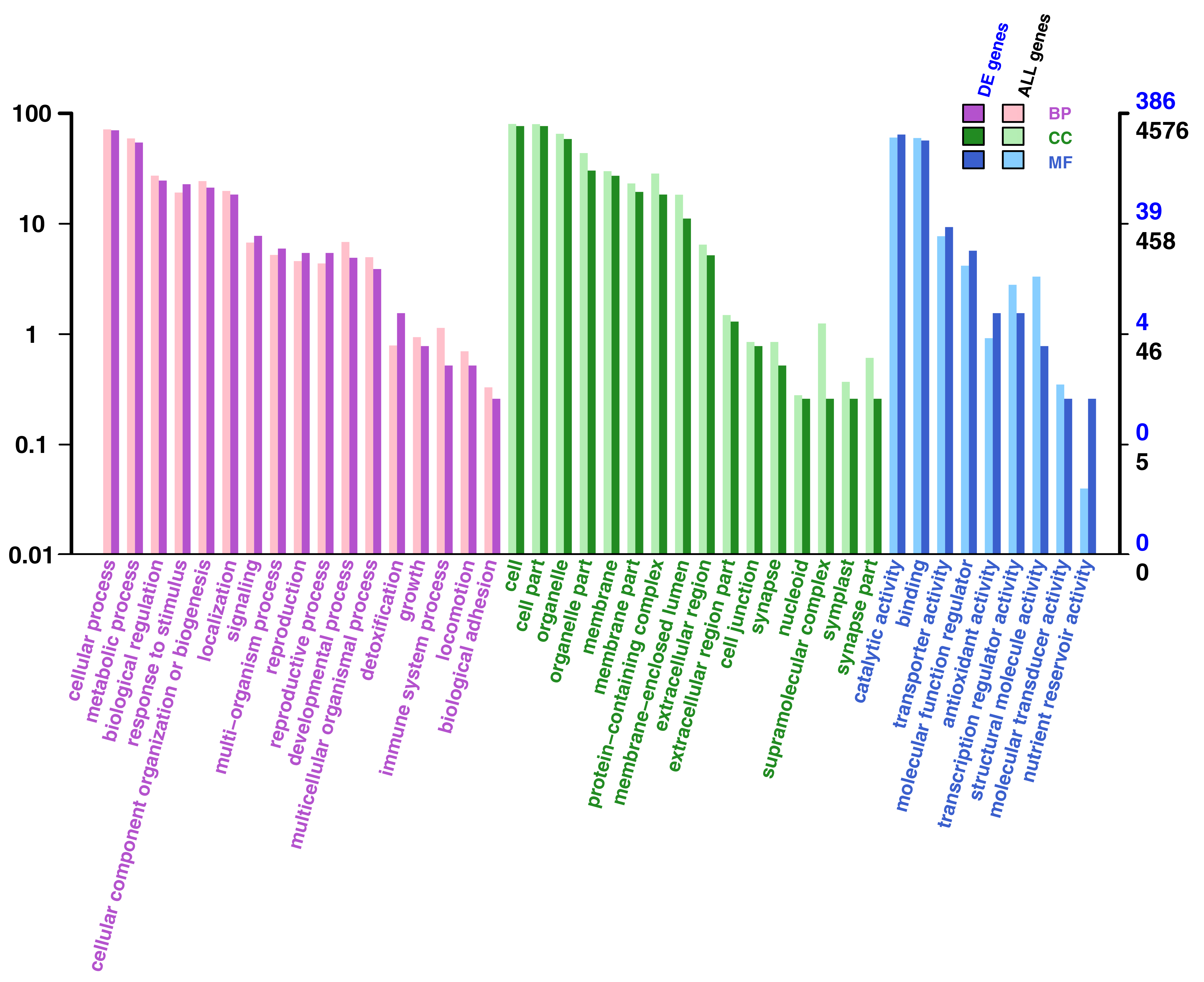

Supplement: Supplementary file 1 [file ijms-26-00849-s001.zip › ijms-3426991-supplementary/Figure. S3.tif]

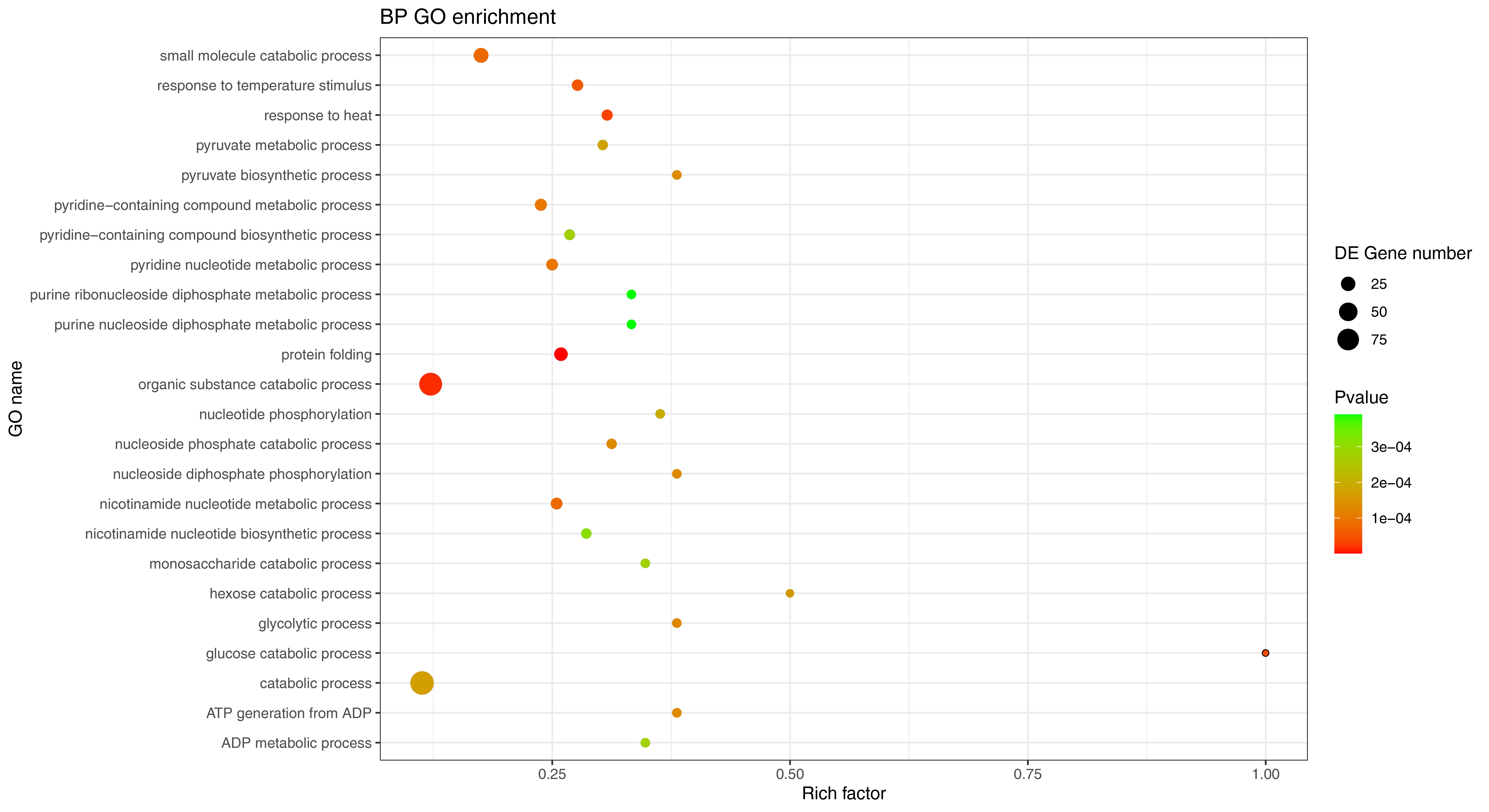

Supplement: Supplementary file 1 [file ijms-26-00849-s001.zip › ijms-3426991-supplementary/Figure. S4.tif]

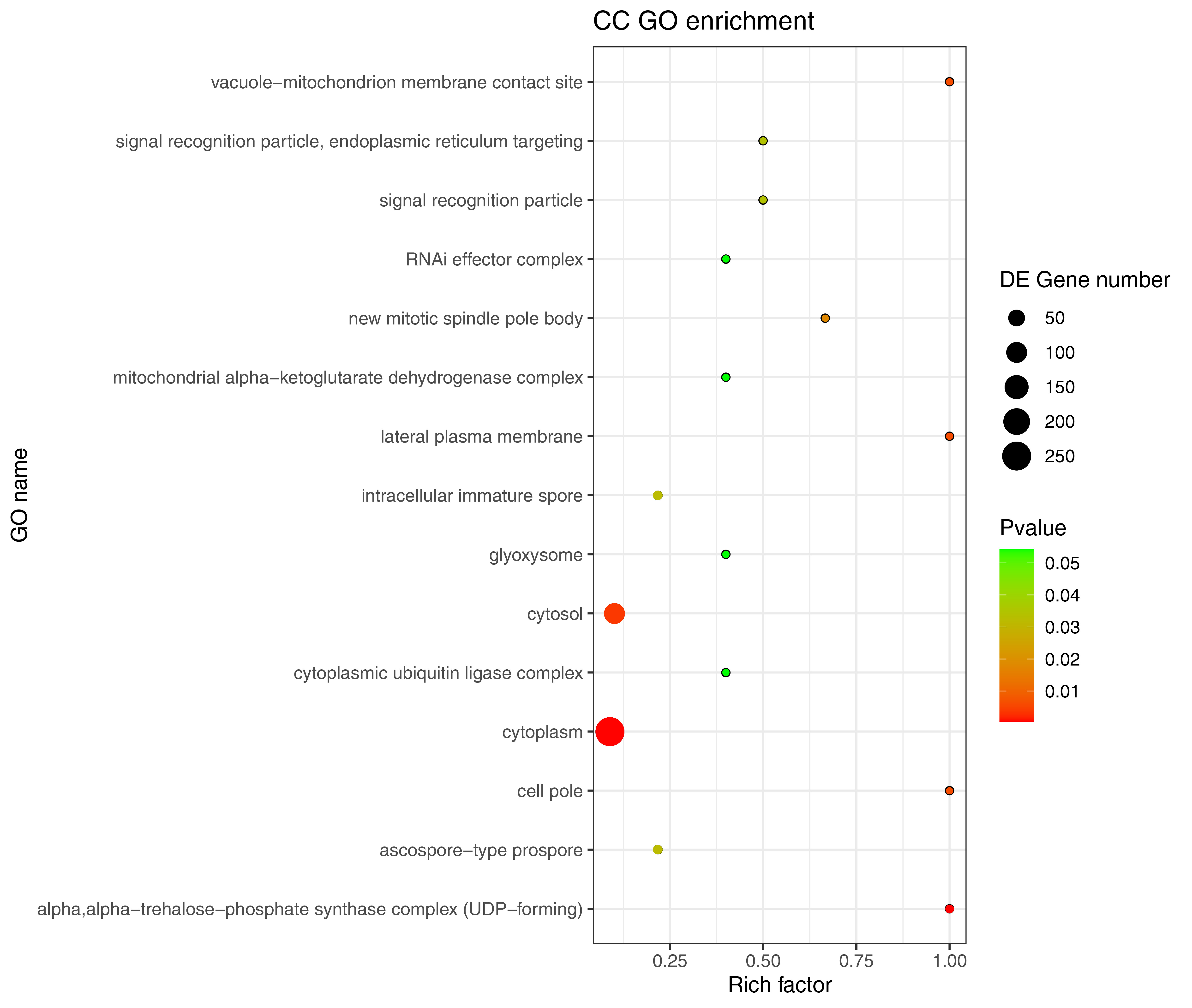

Supplement: Supplementary file 1 [file ijms-26-00849-s001.zip › ijms-3426991-supplementary/Figure. S5.tif]

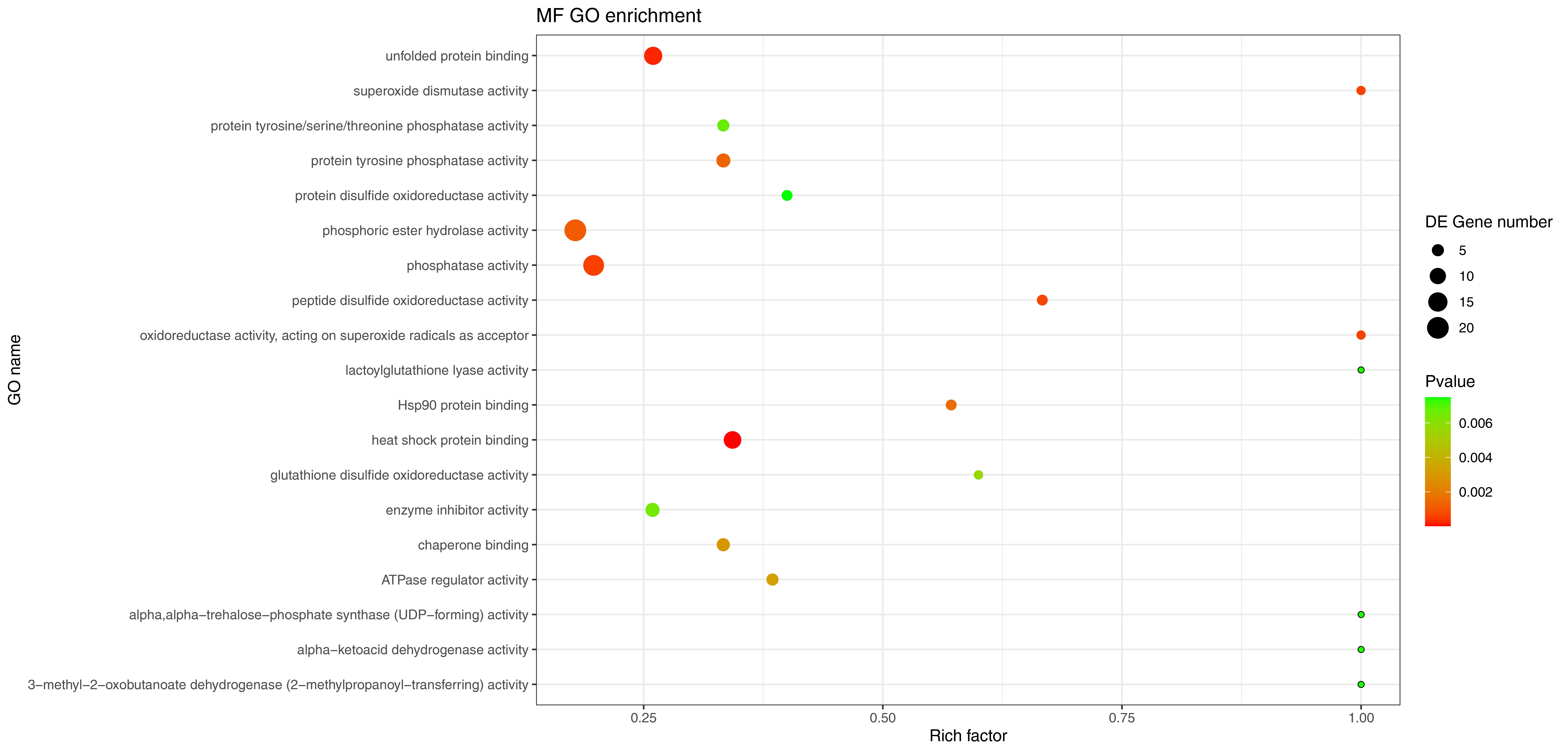

Supplement: Supplementary file 1 [file ijms-26-00849-s001.zip › ijms-3426991-supplementary/Figure. S6.tif]
